# Efficacy of screening for high blood pressure in dental health care

**DOI:** 10.1186/1471-2458-11-194

**Published:** 2011-03-30

**Authors:** Sevek Engström, Christian Berne, Lars Gahnberg, Kurt Svärdsudd

**Affiliations:** 1 Uppsala University, Department of Public Health and Caring Sciences, Family Medicine and Clinical Epidemiology Section, Uppsala, Sweden; 2 Centre for Clinical Research Uppsala University/County Council of Gävleborg, Gävle, Sweden; 3 Public Dental Service, County Council of Gävleborg, Gävle, Sweden; 4 Uppsala University, Department of Medical Sciences, Uppsala, Sweden; 5 University of Gothenburg, Department of Odontology, Gothenburg, Sweden

## Abstract

**Background:**

There is consensus on the importance of early detection and treatment of high blood pressure. Dental care is one of few medical services to which a considerable proportion of the general population comes for regular check-ups. We tested the effects of blood pressure screening in dental care centres with subsequent work-up of subjects screening positive in primary health care (PHCC).

**Methods:**

Altogether 1,149 subjects 40-65 years old or 20-39 years old with body mass index >25, and with no previously known hypertension, who came for a dental examination had their blood pressure measured with an Omron M4^® ^automatic blood pressure reading device. Subjects with systolic blood pressure readings above 160 mmHg or diastolic above 90 mmHg were referred to their PHCC for a check up. Outcome data were obtained by scrutiny of PHCC and hospital patient records for hypertension diagnoses during the three years following screening.

**Results:**

237 (20.6%) subjects screened positive. Of these, 230 (97.1%) came to their PHCC within the 3-year follow-up period, as compared with 695 (76.2%) of those who screened negative (p < 0.0001). Of those who screened positive, 76 (32.1%) received a diagnosis of hypertension, as compared with 26 (2.9%) of those who screened negative. Sensitivity was 79.1%, specificity 84.8% and positive predictive value 30.1%. The number of subjects needed to screen to find one case of hypertension was 18.

**Conclusions:**

Co-operation between dental and primary care for blood pressure screening and work-up appears to be an effective way of detecting previously unknown hypertension.

## Background

Hypertension is a global health problem. It has been estimated that approximately 1.8 million (27%) adults in Sweden may have high blood pressure, according to the World Health Organization criteria [1]. The consequences of non-treated high blood pressure are well known and include increased risk of developing heart disease, stroke, kidney disease and retinopathy [2]. There is a general consensus that the best way to avoid such problems is early detection and treatment of the high blood pressure, before organ damage has occurred.

Many subjects with hypertension are unaware of their condition. Most incidents of hypertension are detected at primary health care, usually *en passant *when patients seek care for other conditions, a kind of non-systematic opportunistic screening.

In industrialised countries, dental care is usually the only public health care organisation to which healthy people come for regular checkups, usually at least once every second year [3,4]. Patients only come to almost all other health care organizations for consultations when they are ill or have a health complaint. Thus the dental service might be one of the most suitable health care organisations for systematic opportunistic screening of healthy subjects as well as for subjects with dental disease. However, one prerequisite for success is a multidisciplinary approach with cooperation between the dental services and primary health care services for clinical work-ups of subjects screening positive [4].

The aim of this cooperative project between the dental and primary healthcare service was to test the efficacy of blood pressure screening in the dental service with subsequent workup in primary care.

## Methods

### Setting

The overwhelming part of Swedish medical care is run by the counties, which have a similar legislative position as the US states. They are responsible for health care within their area, either by County council operated health care units (at the time of the study the vast majority) or by subcontracted private units. Regarding dental health care the situation is similar, except that approximately half of the units are County council operated and the remaining are private units, subcontracted by the central government. However, all units, whether County council operated or private subcontractor, follow the same regulations.

The blood pressure screening study was performed at two County council operated dental clinics located in a small municipality (population 12,000) in the northern part of Gävleborg County, Sweden (population 277,000), approximately 250 kilometres north of Stockholm. The two primary health care centres (PHCC) in the municipality, both County council operated, were partners in the project and were responsible for calibration of the blood pressure reading device used and for blood pressure checkups for patients screening positive.

### Study population

**Figure 1:**
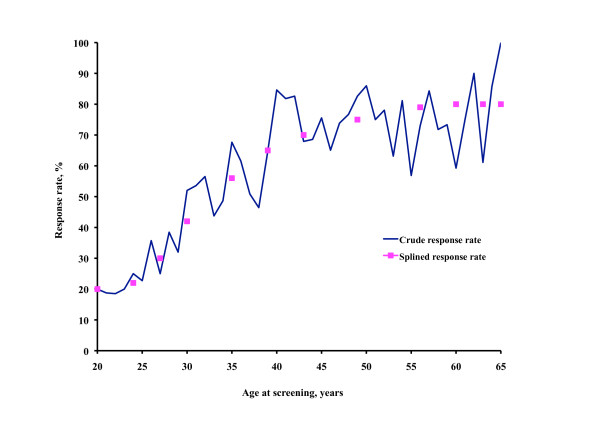
**Screening participation**. Screening participation rate by age.

At the two dental clinics, all consecutive patients aged 20-65 scheduled for a regular check up from 15 May 2003 to 20 December 2005 and living within the municipality were invited by letter to participate in the study. The screening procedure was performed at the dental appointment before the dental examination. Participants were asked to state their height and weight and whether they had a known hypertension. Patients who had no known hypertension and who were in the age range 20-39 with a body mass index (BMI) >25 or in the age range 40-65 regardless of BMI were eligible for screening. Of the 1,791 eligible subjects 1,149 (64%) agreed to have their blood pressure measured. The agreement rate was highly age dependent as shown in Figure 1.

### Data collection

Blood pressure was measured in a sitting position, in the left arm, after 5 minutes' rest, with an automatic blood pressure reading device (Omron M4^®^). If the systolic blood pressure reading was above 160 mmHg or the diastolic above 90 mmHg a second reading was taken after the dental examination and the lowest recorded value was used as the screening blood pressure. Data measured at the dental clinics were registered in pre-prepared protocols and entered into the study database. Subjects with screening systolic blood pressure >160 or a diastolic blood pressure >90 were asked for a referral permission (all accepted). A copy of the dental service protocol was sent as referral document for work up to the subject's PHCC, where an appointment was arranged.

Data on all appointments at the two PHCCs for the complete study population, regardless of screening result, for the three years preceding and the three years following the screening appointment were obtained from the PHCC medical records data base. To check for completeness, appointment logbooks were also scrutinized. Data included appointment date, category of care provider (GP, district nurse, hypertension nurse, physiotherapist, etc.), and for GP and hypertension nurse appointments, diagnoses. The latter were coded according to the International Classification of Diseases [5] and were also given in plain text. In addition, discharge diagnoses after hospital admissions within the three years following screening were scrutinized for hypertension diagnoses. Moreover, the first PHCC blood pressure reading for subjects referred from the dental clinics to the PHCCs was obtained.

Three outcomes were used in this study. The first was whether the referred subjects actually came to the PHCC for follow up, the second whether blood pressure was measured, and the third whether a hypertension diagnosis was established during the first three years after screening. The presence of a hypertension diagnosis during the three years preceding screening was also sought for an additional check of hypertension status at the time of screening.

All participants gave their written informed consent. The study was performed in accordance with the Helsinki Declaration and was approved several times during the data collection process by the Research Ethics Committee at Uppsala University and later by the Regional Research Ethics Board.

### Statistical considerations

Data was analysed using the SAS software, version 9.1 [6]. Summary statistics, such as means and measures of dispersion, were computed with traditional parametric methods. Simple differences between the groups regarding continuous data were tested using Student's t-test or analysis of variance, and nominal or ordinal data with the chi-square test.

The cumulative distribution of first PHCC post-screening follow-up appointment was analysed using Cox's proportional hazards regression, with the first appointment as outcome (dependent) variable and the group variable (screened positive or negative), age and sex as the independent variables. Follow-up time was computed as the number of days from screening until outcome or end of follow up. The subjects were censored at time of death or at end of follow up, whichever came first.

The cumulative distribution of receiving a hypertension diagnosis based on PHCC and hospital records among subjects with no such diagnosis at screening was analysed accordingly, with first hypertension diagnosis as outcome and the group variable (screening positively or negatively), age, sex, and screening systolic and diastolic blood pressure as independent variables.

The numbers needed to screen (NNS) to find a new case of hypertension, a parallel to numbers needed to treat (NNT) in randomised clinical drug trials, was computed in a similar way to NNT, as the reciprocal of the proportion of new cases found by screening, over and above those who would have been detected in any case [7]. The numbers used are shown in the Results section. All tests were two-tailed. A p-level of less than 0.05 was set to indicate significance.

## Results

### Characteristics of the study population

Some characteristics of the study population are given in Table 1. Half the population was female, mean age at screening was 46 years, mean height 173 centimetres, mean weight 79 kilograms, and mean body mass index 26. During the three years preceding screening, 861 subjects, 74.9% of the 1,149 eligible subjects, had seen their GP at least once, on average 3.2 times per subject. Moreover, one (0.01%) subject had been admitted to hospital once. During the three years following screening 925 (80.5%) subjects saw their GP at least once, on average 3.9 times per subject, and six (0.5%) subjects had altogether 12 admissions to hospital. Seven (0.6%) subjects died during follow up.

**Table 1:** Characteristics of the study population

	N	Mean (SD) or %
No. of eligible patients	1,149	100
Mean age at screening, years	1,149	46.4 (9.52)
Women, %	573	49.9
Reported height, cm	1,149	172.7 (9.23)
Reported weight, kg	1,149	78.8 (14.78)
Body mass index, kg/m^2^	1,149	26.3 (3.92)
Three-year period prior to screening		
GP appointments	3,639	
Patients seen by GP	861	74.9
Hospital admissions	1	
No. of patients admitted	1	0.1
Three-year period following screening		
GP appointments	4,309	
Patients seen by GP	925	80.5
Hospital admissions	12	
No. of patients admitted	6	0.5
Deceased after dental screening appointment	7	0.6

**Figure 2:**
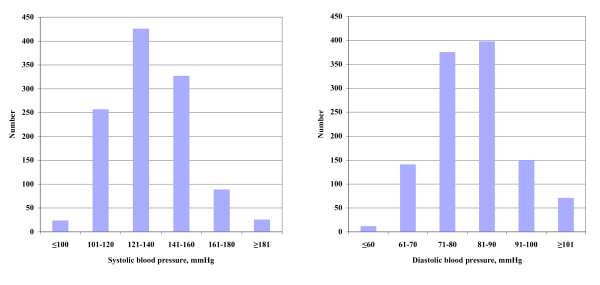
**Screening blood pressure distribution**. Distribution of screening systolic blood pressure and diastolic blood pressure.

The distribution of the screening systolic and diastolic blood pressure is shown in Figure 2. The systolic blood pressure range was 84-223 mmHg, mean 135 mmHg, median 133 mmHg. The corresponding values for diastolic blood pressure were 44-129 mmHg, 82 mmHg and 81 mmHg.

### Screening outcome

Out of the 1,149 subjects, 115 (10.1%) had screening systolic blood pressure >160 mmHg, and 221 (19.2%) had diastolic blood pressure >90 mmHg, Table 2. In all, 237 (20.6%) had systolic or diastolic blood pressure above the cut-off point, and were therefore referred to their PHCC. Of these subjects, 221 (93.2%) had no hypertension diagnosis in the PHCC records or hospital discharge data during the three years preceding the index dental service appointment, five (2.1%) had been subjected to a blood pressure work-up but no hypertension diagnosis was arrived at, and 11 (4.6%) had a previous hypertension diagnosis which they denied on occasion of screening.

**Table 2:** Results of the screening procedure and follow up

	Screening result			
	
	Negative		Positive	
	
	n	%	n	%
Systolic blood pressure >160 mmHg	1,034	89.9	115	10.1
Diastolic blood pressure >90 mmHg	928	80.8	221	19.2
Systolic >160 mmHg or diastolic >90 mmHg	912	79.4	237	20.6
Hypertension history				
No previous hypertension diagnosis	892	97.8	221	93.2
Previous hypertension work-up but no diagnosis	5	0.5	5	2.1
Previous hypertension diagnosis	15	1.6	11	4.6
Subjects who saw their GP within 3 years	695	76.2	230	97.1
Blood pressure measured	-*^)^	-	230	97.1
No hypertension diagnosis	872	95.6	84	35.4
Hypertension work-up, no diagnosis	14	1.5	77	32.5
Hypertension diagnosis established	26	2.9	76	32.1

*^) ^data not available

During the three years following screening 230 (97.1%) of the 237 subjects referred saw a district nurse or their GP, and had their blood pressure measured. The corresponding numbers among the non-referred was 695 (76.2%), p for difference < 0.0001.

Of the referred subjects 84 (35.4%) did not receive diagnosis of hypertension, 77 (32.5%) were subjected to a hypertension work-up but received no hypertension diagnosis, and 76 (32.1%) received a hypertension diagnosis. The corresponding numbers for those not referred were 872 (95.6%), 14 (1.5%), and 26 (2.9%). The difference in work-up result between referred and non-referred subjects was highly significant (p < 0.0001).

Of the 76 subjects who received a hypertension diagnosis during follow up, 66 (86.8%) had no previous history of hypertension, two (2.6%) had a previous work up performed but no diagnosis, and eight (10.5%) had a previous diagnosis of hypertension.

**Figure 3:**
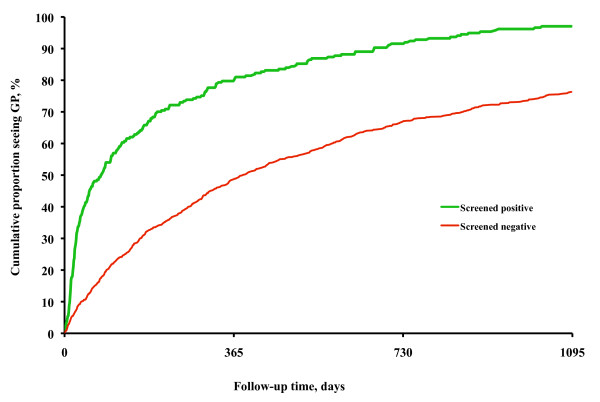
**Proportions seeing GP**. Cumulative percentage of those who screened positive and negative who saw their general practitioner during three years of follow up.

In Figure 3 the cumulative distribution of first PHCC follow-up appointments by day after the screening is shown for those referred and those not referred. The cumulative proportion of subjects seeing their GP increased much more rapidly among referred subjects than among not referred during the first 180 days. After this time period the rate of increase was about the same in the two groups, even though it occurred on a higher level among referred. The tendency to see the GP was affected by referral (HR 2.55, 95%CI 2.18-2.98, p < 0.0001), by age (HR 1.02, 95%CI 1.01-1.02, p < 0.0001), and by sex (HR men to women 0.88, 95%CI 0.77-0.998, p < 0.05).

**Figure 4:**
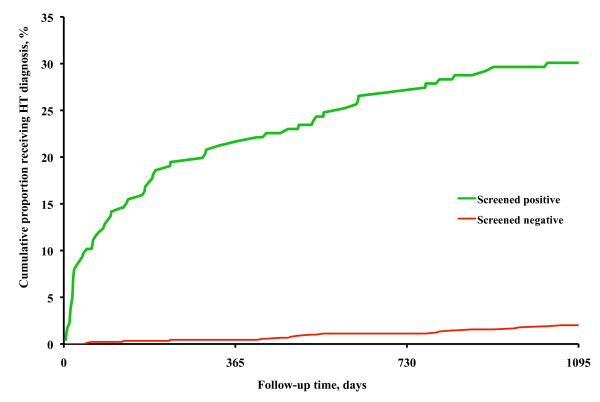
**Proportions being diagnosed**. Cumulative percentage of those who received a diagnosis of hypertension during three years of follow up.

The cumulative proportion of subjects who received a diagnosis of hypertension among those who had no diagnosis at screening is shown in Figure 4. Among the referred subjects, the proportion with a diagnosis of hypertension increased rapidly during the first few months after the dental appointment, then levelled off. Among the non-referred the proportion increased slowly and successively during the three-year follow up. The probability of receiving a diagnosis of hypertension was affected by age (HR 1.02, 95%CI 1.001-1.02), systolic and diastolic screening blood pressure (HR 1.03, 95%CI 1.02-1.04, and 1.08, 95%CI 1.06-1.10, respectively), but not by sex (HR men to women 0.90, 95%CI 0.67-1.22). Of those who received a diagnosis 59.2% were men, on average 51.9 (SD 6.94) years old, had a screening blood pressure of 169.9 (SD 18.03)/101.8 (SD 9.58) mmHg, and a first PHCC blood pressure reading of 154.1 (SD 19.89)/89.2 (SD 12.14) mmHg. The corresponding data for those who did not receive a diagnosis were 60.9%, 48.1 (SD 9.14) years, 156.1 (SD 13.54)/96.2 (SD 6.11) mmHg, and 136.6 (SD 12.57)/80.6 (SD 7.71) mmHg.

Of the 86 previously unknown cases of hypertension found during follow up, 68 screened positive, yielding a sensitivity of 79.1%, Table 3. Of the 1,037 subjects who did not receive a diagnosis of hypertension during follow up 879 screened negative, giving a specificity of 84.8%. Of the 226 with no previous diagnosis of hypertension and who screened positive, 68 received a diagnosis of hypertension, yielding a positive predictive value of 30.1%. A Receiver Operating Characteristics curve (ROC) analysis showed that the optimal systolic blood pressure screening level would have been 145-150 mmHg and the diastolic level 85-90 mmHg.

**Table 3:** Data for sensitivity, specificity, and predictive value calculations

	Work-up result		
	
	No hypertension diagnosis	Hypertension diagnosis	Total
Screening result			
Negative	879	18	897
Positive	158	68	226

Total	1,037	86	1,123

Among subjects who screened negative, 18 out of 897 (2.0%) received a hypertension diagnosis during follow up, Table 3. Assuming the same detection rate among all the subjects screened would have given approximately 23 (2% of 1,149 subjects) subjects with new diagnosis of hypertension if no screening had been performed. With screening, 63 (86-23) new hypertensive patients were identified, over and above those who would have been detected in any case. NNS based on these assumptions would then be 1/(63/1,149) = 18.2 screened to find one case.

## Discussion

In this co-operative study between the dental and primary health care services every fifth patient who came for a regular dental examination and an opportunistic blood pressure screening had systolic blood pressure >160 mmHg or diastolic blood pressure >90 mmHg. Of those who screened positive almost all saw their GP for a blood pressure follow up, and one third received a diagnosis of hypertension within three years of screening. Screening sensitivity was 79%, specificity 85%, positive predictive value 30%, and NNS was 18.

The conditions during the blood pressure screening performed in this study were basically the same as in blood pressure screening performed by medical care practitioners. The blood pressure measurement procedure was standardized, with all measurements in the left arm after at least five minutes' rest. It has previously been shown that the blood pressure level falls during the first few minutes of rest but has attained a stable level within five minutes, irrespective of blood pressure level [8,9]. The Omron M4^® ^device has been tested against manual blood pressure readings. The variability of the readings appears to be comparable to that for manual readings performed by different observers [10].

The strengths of this study include the fact that the study was performed as opportunistic screening at the dental care service, the only medical service that sees a considerable proportion of the general public annually or biannually, yielding a large study population at low cost. The co-operation with the primary care services was a prerequisite for a successful work-up of subjects who screened positive. Other strengths include the close monitoring of outcome, i.e., a diagnosis of hypertension in subjects screened during follow up, with an almost 100% complete information.

The limitations include scanty clinical information from the GP appointments, such as the hypertension diagnosis criteria. However, we have reason to believe that the GPs were following the national guidelines for diagnosing high blood pressure, based on World Health Organization (WHO) recommendations, or used somewhat higher hypertension criteria [11]. This means that the results of this study are minimum results in the sense that diagnostic procedures carried out strictly according to guidelines might have yielded even better results.

It is well known that screening blood pressures tend to be higher than pressure readings at clinics, which in turn are higher than blood pressures measured at home [12,13]. This is known as so called white coat hypertension and was not caused by erroneous screening blood pressure readings. The equipment was regularly checked by the co-operating PHCCs. In spite of the tendency to have higher readings at screening than during work up we chose to use the blood pressure levels indicating hypertension as proposed by the Fourth Joint European Societies Recommendation on Prevention of Coronary Heart Disease in Clinical Practice guidelines [14], anticipating a considerable proportion of "false positives" or "noise", i.e., subjects screening positive owing to temporarily high blood pressure readings.

In this case, 70% of subjects who screened positive were false positives in the sense that they did not receive a hypertension diagnosis during the three-year follow up. Using a higher cut-off blood pressure level would have yielded fewer false positives and higher specificity but lower sensitivity. In this study we chose a cut-off level that turned out to be close to the optimum one, as determined in the ROC curve based analysis.

The risks associated with high blood pressure in terms of developing various forms of cardiovascular disease are well known and have been summarized in the Fourth Joint European Societies Recommendation on Prevention of Coronary Heart Disease in Clinical Practice guidelines [14]. The general idea of the guidelines is that, all things considered, early detection and treatment of high blood pressure is associated with a better outcome than if the condition is detected late in its course.

Traditionally blood pressure screening, especially in connection with scientific studies, has been performed by special organisations. However, the participation rate is a moderate 60%-67% and the cost of screening is high [15]. The alternative, opportunistic screening, involves using an existing organisation with screening cost being marginal in relation to the total cost of the organisation. Opportunistic screening with subsequent medical care should preferably be performed by the primary care service, where patient selection is low. However, the screening must be long-term, each screening round taking up to five years to screen 80% or more of the general population [16]. In the present study the mean participation rate was 64%, but in the age group of most interest in blood pressure screening, 40 years of age or older, the participation rate was 70%-80% during the three-year screening period, as shown in Figure 1.

The results of screening carried out at other types of facilities, such as supermarkets [17,18] and pharmacies [19] have been reported as successful in terms of the number of people screened (numerator), but may imply problems, such as handling of confidential information, and the uncertainty of the size and geographical delineation of the underlying general population (denominator). By screening at the dental care services, as in the present study, a number of these problems were avoided. Dental care is one of few medical services to which a considerable proportion of the general population comes for regular check-ups. In Sweden more than 80% of the general population are seen by the dental care services within a two-year period [3], there are no problems with handling of confidential information, and the underlying general population can easily be determined. In all screening activities, subjects who screen positive must be followed up. In this study, the follow up was carried out at the primary health care services, the cooperating partner. This partnership was one of the prerequisites for success.

Even though the study was performed in Sweden, the results appear to be applicable to all geographical areas with a similar structure of medical and dental services, for instance the Nordic countries and the United Kingdom.

## Conclusions

In this co-operative project between dental and primary care services the blood pressure screening procedure was efficient. One fifth screened positive, the overwhelming majority of those who screened positive came for follow up, and one third of those who screened positive received a diagnosis of hypertension during the three-year follow up. On average, for every 18 subjects screened one case of hypertension was found. The procedure therefore appears to be an effective way of detecting unknown hypertension.

## Competing interests

The authors declare that they have no competing interests.

## Authors' contributions

SE and KS designed the study. SE was responsible for and supervised the data collection. SE and KS performed the data analyses, and drafted the manuscript. All authors participated in the discussions of the results, provision of reference literature and in the manuscript revisions. All authors have seen and approved the final manuscript version.

## Pre-publication history

The pre-publication history for this paper can be accessed here:

http://www.biomedcentral.com/1471-2458/11/194/prepub
